# Bioactivities of Phenolics by Focusing on Suppression of Chronic Diseases: A Review

**DOI:** 10.3390/ijms19061573

**Published:** 2018-05-25

**Authors:** Fereidoon Shahidi, JuDong Yeo

**Affiliations:** Department of Biochemistry, Memorial University of Newfoundland, St. John’s, NL A1B 3X9, Canada; jy2402@mun.ca

**Keywords:** phenolics, bioactivity, phenolic acid, flavonoids, anticancer, anti-inflammatory activity, antibacterial activity

## Abstract

Phenolics, which are secondary metabolites of plants, exhibit remarkable bioactivities. In this contribution, we have focused on their protective effect against chronic diseases rather than their antioxidant activities, which have been widely discussed in the literature. A large body of epidemiological studies has proven the bioactivities of phenolics in both standard compounds and natural extracts: namely, anticancer, anti-inflammatory, and antibacterial activities as well as reducing diabetes, cardiovascular disease, and neurodegenerative disease. Phenolics also display anti-analgesic, anti-allergic, and anti-Alzheimer’s properties. Thus, this review provides crucial information for better understanding the bioactivities of phenolics in foods and fills a gap in the existing collective and overall knowledge in the field.

## 1. Introduction

### 1.1. Phenolics

Phenolic compounds are secondary metabolites of plants. So far, more than 8000 phenolics have been found from natural sources and are classified into phenolic acids, flavonoids, stilbenes, coumarins, lignins, and tannins. Phenolics play a crucial role in plants by controlling their growth as an internal physiological regulator [1]. For instance, kaempferol, apigenin, and quercetin interact with plasma membrane proteins (receptors), in which they restrict the transfer of polar auxin compounds via the membrane, thus affecting plant growth [1]. Phenolics in the outer part of plants shield them from fatal high-energy wavelengths by absorbing them in advance; in other words, electron-rich parts such as the π-bond of the phenolics absorb the wavelength before attacking the critical parts of the cells.

### 1.2. Classification of Phenolics

**Figure 1:**
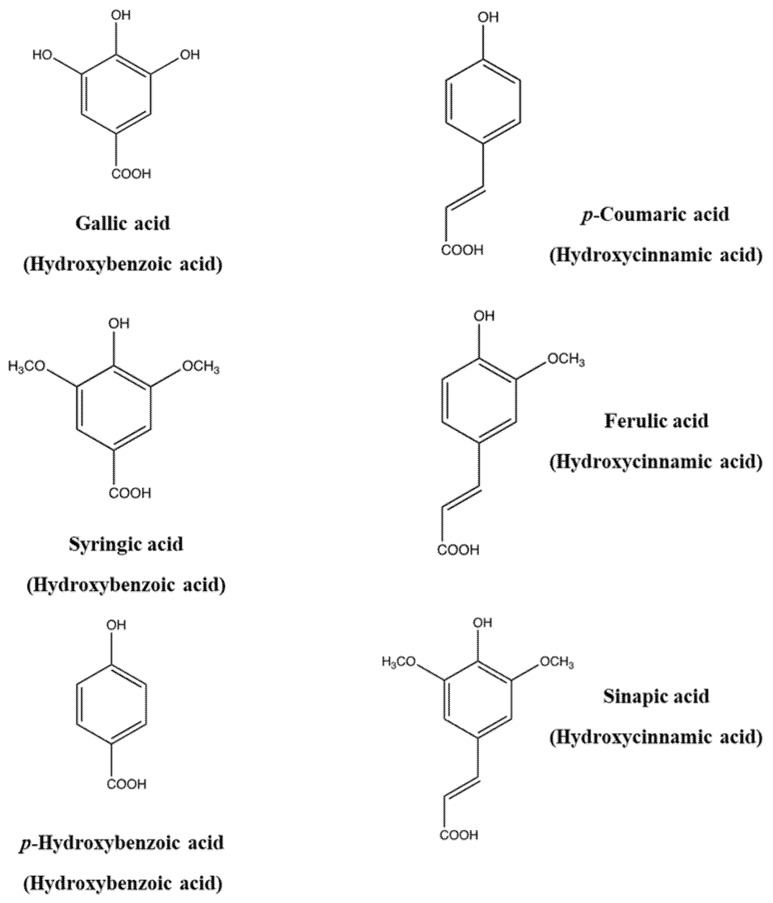
Chemical structures of representative phenolic acids.

Phenolic and polyphenolic compounds can be classified into several classes, namely phenolic acids, flavonoids, stilbenes, coumarins, lignins, and tannins. Phenolic acids constitute a primary phenolic class of compounds in natural sources such as cereals, legumes, and other seeds, in which they act as the building material of cell wall matrices by forming bridges with macromolecules such as cellulose, hemicellulose, and pectin, thus supporting the construction of compact cell wall structures. Thus, they generally occur in various conjugated forms other than the free type. Phenolic acids are divided into two groups, hydroxycinnamic acids and hydroxybenzoic acids. Hydroxycinnamic acids include *p*-coumaric, caffeic, ferulic and sinapic acids, while hydroxybenzoic acids encompass *p*-hydroxybenzoic, protocatechuic, vanillic, syringic and gallic acids (Figure 1). The differentiation is in the substitution and functional groups, namely hydroxyl and methoxy groups, which decide the individual differences of each phenolic acid. The ingested phenolic acids are absorbed in the gastrointestinal tract and then circulate in the blood system after methylation, sulfation, and glucuronidation in the liver [2]. For example, hydroxycinnamic acids in wines are absorbed in the gastrointestinal tract after ingestion and transformed into glucuronide and sulfate conjugates, followed by circulation in the blood [3]. This conjugation process increases the hydrophilicity of the phenolic compounds and helps remove them via the biliary or the urinary route.

Flavonoids, another class of phenolics that have a three-ring structure in the C6–C3–C6 form, constitute different sub-classes of compounds such as flavones, flavonols, flavanones, flavanonols, flavanols, isoflavones, and anthocyanidins. These compounds may have different substitution patterns with hydroxyl and methoxy groups. Flavonoids possess remarkable bioactivities such as anticancer, anti-inflammation and anti-virus effects as well as reducing cardiovascular diseases and type-2 diabetes, which will be mainly discussed in this contribution. 

Stilbenes, also known as 1,2-diphenylethene, possess a C6–C2–C6 carbon skeleton and show potent bioactivities such as antibacterial effects, among others. For example, tetrastilbenes such as kobophenol-A and -B display antibacterial activity against *Staphylococcus aureus* [4]. Stilbenes are also known as an active inhibitor of topoisomerase II, which causes unwinding of coiled DNA during cellular transcription [5]. Resveratrol, which is abundant in grapes and wines, is a representative example of stilbenes and has shown many bioactivities such as anti-inflammation, anti-tumorigenesis, and cardioprotective effects [6]. In addition, resveratrol is useful in the inhibition of Parkinson’s and Alzheimer’s diseases by moderating hemeoxygenase activity because the incorrect functioning of hemeoxygenase causes such ailments [7,8].

**Figure 2:**
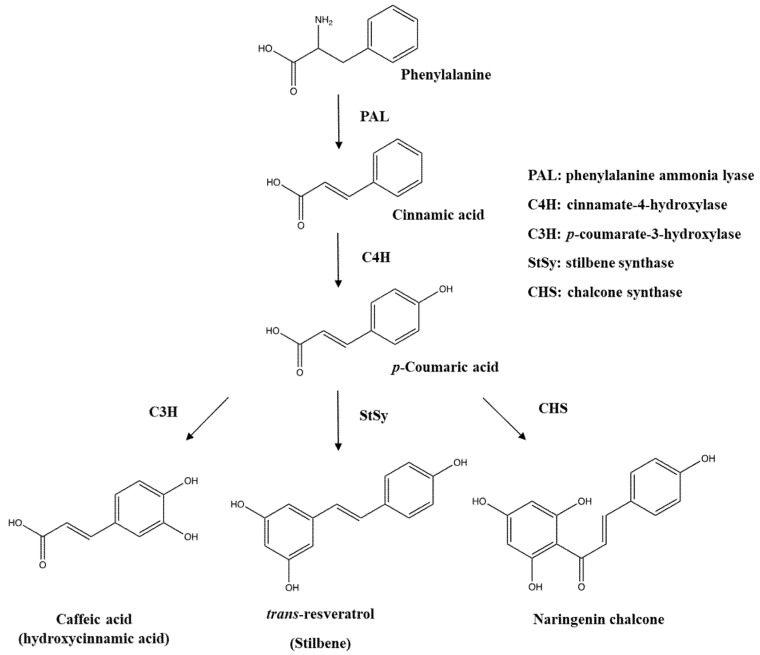
Biosynthesis pathway of phenolic acids and stilbene from phenylalanine.

Coumarins are referred to as benzopyrones with a C6–C3 skeleton and an oxygen heterocycle in the C3 unit, as shown in Figure 2 [9]. They are found in a wide variety of plants such as tonka bean (*Dipteryx odorata*), sweet woodruff (*Galium odoratum*), sweet grass (*Hierochloe odorata*), deertongue (*Dichanthelium clandestinum*), vanilla grass (*Anthoxanthum odoratum*), mullein (*Verbascum* spp.), and sweet-clover (*Melilotus* sp.). Aside from plants, coumarins are present as the metabolites of microorganisms such as *Streptomyces* and *Aspergillus* species, namely novobiocin and coumermycin [9,10].

Coumarins show a variety of bioactivities such as anti-inflammatory, antimicrobial, antiviral, antioxidant, antitumor, antiasthmatic, antidepressant, anti-HIV, and anti-Alzheimer’s [11,12,13,14]. In addition, coumarin possesses anti-coagulant activity, which inhibits blood coagulation by suppressing the hepatic biosynthesis of vitamin K-dependent coagulation factors. Thus, coumarin is also used as an anticoagulant for the treatment of diseases such as thrombotic phlebitis and pulmonary embolism.

Moreover, phenolic compounds may occur in the polymeric type in plants; lignins are the polymers of monolignols such as *p*-coumaric acid and sinapic acid. Tannins are divided into two groups: hydrolyzable tannins such as ellagitannins and gallotannins; and condensed tannins, also referred to as proanthocyanidins. The latter group is further divided into several sub-classes depending on the type of linkage between the flavonoids, namely single or double linkages. Proanthocyanidins are widely distributed in plant-based foods such as lentils, grape seed, cocoa seeds, and apples, among others [15,16,17]. The structural properties of proanthocyanidins, especially those with two or three hydroxyl groups in the B-ring of flavan-3-ol unit, confer bioactivity; for instance, proanthocyanidin shows not only inhibitory activity against the oxidation of low density lipoprotein (LDL) cholesterol, stomach mucosa injury, and radioprotective effects against chromosomal damage, but also a practical bacterial anti-adhesion effect [18,19]. In addition, they possess a robust antioxidant potential such as oxygen radical scavenging capacity [20].

### 1.3. Biosynthesis of Phenolics

The biosynthesis of phenolics takes place at the surface of the endoplasmic reticulum (ER) in plant cells [21]. Phenylalanine and tyrosine are precursors for the biosynthesis of different classes of phenolics such as phenolic acids, flavonoids, stilbenes, coumarins, lignins, and tannins. A variety of enzymes including erythrose-4-phosphate, phenylalanine ammonia lyase (PAL), cinnamate-4-hydrolxylase, *p*-coumarate-3-hydroxylase, and *o*-methyl transferase are involved in the biosynthesis of phenolics [22]. Phenylalanine, which is synthesized via the shikimate pathway, and to a lesser extent tyrosine, are the primary precursors for the biosynthesis of different classes of phenolics. The enzymatic biosynthesis of phenolics begins with releasing an ammonia group of phenylalanine by phenylalanine ammonia lyase (PAL), and this step yields trans-cinnamic acid with the creation of a double bond (Figure 2). The produced trans-cinnamic acid is modified into *p*-coumaric acid by cinnamate-4-hydrolxylase, and subsequently, the assistance of *p*-coumarate-3-hydroxylase generates caffeic acid from *p*-coumaric acid after introducing a hydroxyl group to the aromatic ring. This *p*-coumaric acid is altered into ferulic acid by the action of *o*-methyl transferase, and so ferulic acid is converted into sinapic acid; two enzymes, hydroxylase and *o*-methyl transferase, are involved in the substitution of a methoxy group for the biosynthesis of sinapic acid. Tyrosine, another precursor, is also transformed into *p*-coumaric acid by detaching an ammonia group through the enzymatic reaction of tyrosine ammonia lyase and then *p*-coumaric acid is changed into different types of phenolic acids through the same reactions. In addition, benzoic acid is formed by losing two carbon atoms from phenylpropanoids.

Flavonoids’ biosynthesis, referred to as the flavonoid branch pathway, starts with a combination of three molecules of malonyl coenzyme A (CoA) and *p*-coumaroyl CoA, leading to the formation of naringenin chalcone with the aid of chalcone synthase (CHS), followed by alteration into flavonone by chalcone isomerase. The produced flavonone is converted into different classes of individual flavonoids such as flavone, flavonol, flavononol, flavanone, isoflavone, and anthocyanins under suitable enzymatic reactions. For instance, flavonone is transformed into kaempferol and apigenin by flavonol synthase/flavanone-3-hydroxylase and flavone synthase as well as the production of genistein by isoflavone synthase.

### 1.4. Transfer of Phenolics

The phenolics synthesized in the ER transfer to other organs via membrane transporter proteins and vesicles. The phenolics secreted from the ER after biosynthesis move to the target organs and enter them through ABC (adenosine tri-phosphate (ATP) binding cassette) and MATE (multidrug and toxic compound extrusion) transporter proteins at the surface of the membrane. Phenolics also transfer to other organs through the vesicles. Vesicles, which are small sacs enclosed by a lipid bilayer, can contain liquids and essential molecules, and transport these compounds from one site to another throughout the cells. In the same way, the vesicles incorporate phenolics and transport them to target organs. A large proportion of the phenolics synthesized are transferred to the vacuole, in which they co-exist with other organic acids; approximately 80% of phenolics are localized in the vacuole. Meanwhile, some phenolics transfer to the cell wall matrix and form covalent bonds such as ester, ether, and C–C bonds with cell wall substances (e.g., cellulose, pectin, arabinoxylan, and structural proteins). These phenolics are referred to as insoluble-bound phenolics since they are not isolated by the commonly used extraction media due to the covalent bonds to the insoluble macromolecules. 

### 1.5. Absorption of Phenolics in the Digestive Tract

Phenolics from plant-based foods undergo multi-enzyme reactions in the digestive tracts such as the mouth, stomach, small and large intestines (colon) after consumption. Phenolics in food matrix are biologically extracted in the gastrointestinal tract by different enzymes and pH conditions [23]. The phenolics liberated from the food matrix are absorbed in the small intestine. Once they pass through the epithelial cells, they simultaneously conjugate with other molecules such as glucuronic acid and sulfonate, followed by introduction in the plasma [24]. However, the small intestine absorbs only a small portion of free phenolics in the food matrices and only 5–10% of them are incorporated into the plasma; the remaining 90–95% are directly transferred to the colon [21]. The phenolics reaching the large intestine undergo fermentation by the versatile colon microbiota [25,26]. In the colon, approximately 1000 different microorganisms such as bacteria, fungi, protozoa, and archaea co-exist and take part in the elaborate fermentation process of unabsorbed material, including phenolics [27]. These microorganisms secrete extracellular enzymes, thus affecting the chemical structure of phenolics. The phenolics existing in the colon have a positive effect on health by reducing the pH of the colon, which inhibits the growth and proliferation of cancer-inducing microorganisms; for instance, phenolics from blueberries suppress the growth of colon cancer cell lines such as HT-29 and Caco-2 by approximately 50% [28]. 

Epidemiological evidence has so far shown many health-promoting actions of the absorbed phenolics such as anticancer, anti-inflammatory, and anti-diabetes. This review discusses a variety of bioactivities of standard phenolics and phenolic extracts from natural origins such as grains, cereals, legumes, seeds, and fruits by focusing on their preventive effects against chronic ailments and summarizing clinical evidence.

## 2. Bioactivities of Standard Phenolics

### 2.1. Anticancer

Epidemiological evidence has demonstrated the remarkable health-promoting effects of phenolics on chronical ailments, including anti-carcinogenic, anti-inflammatory, and antioxidant activities (Table 1). The anti-carcinogenic capacity is a primary disease-preventive effect of phenolics; they retard the initiation and progression of cancers by constraining the transformation of normal cells, the growing tumors, angiogenesis, and metastasis. Moreover, phenolics stimulate the expression of tumor-suppressing proteins such as p53, phosphatase and tensin homolog (PTEN), p21, and p27 [29]. Gallic acid has shown significant anticancer activity in many studies by attenuating the growth of different types of cancer cells such as human leukemia (HL)-60 and DU-145 human prostate carcinoma cells [30,31]. Methyl gallate also suppresses the proliferation of human epidermoid carcinoma (A431) skin cancer cells [32]. The anticarcinogenic potential of other phenolic acids such as ferulic, feruloyl-l-arabinose and coumaric has also been investigated in several cell lines. Fahrioğlu et al. [33] reported that ferulic acid shows an anticancer effect by influencing the cell cycle, invasion, and apoptotic behavior of MIA PaCa-2 (human pancreatic cells). Eitsuka et al. [34] studied the synergistic anticancer potential of ferulic acid and δ-tocotrienol against the proliferation of different cancer cells in which they found that this combination displays a better inhibitory effect on the proliferation of DU-145 (prostate cancer), michigan cancer foundation-7 (MCF-7, breast cancer), and PANC-1 (pancreatic cancer) cells compared to their individual use. Choi and Park [35] found that ferulic acid inhibits homologous recombination (HR) repair of DNA and RAD 51 (eukaryotic gene) formation in breast cancer cells. Moreover, ferulic acid revealed a remarkable chemotherapeutic effect in combination with veliparib treatment. Janicke et al. [36] noticed the protective effect of *p*-coumaric acid against the development of colon cancer by retarding the cell cycle progression of Caco-2 colon cancer cells. Feruloyl-l-arabinose suppressed penetration, migration, and production of reactive oxygen species (ROS) in lung cancer cells [37]. Furthermore, flavonoids such as troxerutin, apigenin, kaempferol, and myricetin have proven their excellent anticarcinogenic capacity. Panat et al. [38] studied the binding affinity of troxerutin to the DNA groove in order to induce cytotoxicity of prostate cancer cell using γ-radiation, and the result revealed an effective inhibition of the proliferation of that cancer cell. Apigenin displayed a radiosensitizing effect in human tumor cells in which the cancer cell treated with apigenin showed a higher radiosensitivity and apoptosis levels than that without apigenin [39]. Leung et al. [40] explored the apoptosis-inducing potential of kaempferol in human lung carcinoma cells, and they argued that the apoptosis of aforementioned cancer cell by kaempferol may be due to the alteration in apoptotic markers including caspase-3 (caspase-dependent) and apoptosis-inducing factor (AIF, caspase-independent). In addition, myricetin showed a strong anticancer capacity in 1,2-dimethylhydrazine-induced carcinogenesis in colorectal cancer of rat [41]. 

### 2.2. Anti-Inflammatory Activity

**Figure 3:**
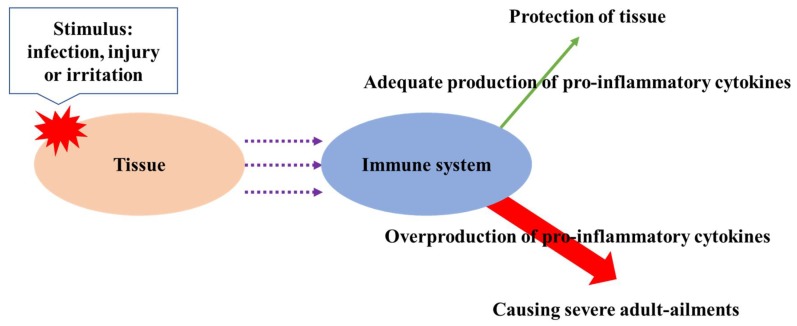
The occurrence of chronical diseases by the overproduction of pro-inflammatory cytokines.

Inflammation is an essential biological response to tissue damage. The immune system responds to stimuli such as infection, injury, or irritation through the release of pro-inflammatory cytokines [58]. The overproduction of pro-inflammatory cytokines such as interleukin (IL)-1b, IL-6, and tumor necrosis factor alpha (TNF-α) cause severe adult ailments such as arthritis, allergy, atherosclerosis, and cancer (Figure 3) [52]. Thus, inhibition of overproduction of pro-inflammatory cytokines is vital in order to prevent related ailments. Phytochemicals from plant-derived formulations have been extensively used to treat inflammation and related disorders from the ancient time. Phenolics are essential compounds for the suppression of inflammation among phytochemicals, and the recent literature has revealed their potent anti-inflammatory capacity. Pragasam et al. [59] studied the anti-inflammatory effects of *p*-coumaric acid by monitoring the expression of tumor necrosis factor (TNF-α) in synovial tissue of adjuvant-induced arthritic rats, and the potent anti-inflammatory activity of *p*-coumaric acid was found by lowering the expression of inflammatory mediator TNF-α. Anti-inflammatory activity of caffeic acid and ellagic acid was also found by Chao et al. [60]; caffeic and ellagic acids were supplied to mice by mixing to their diet at a ratio of 2.5 and 5.0%, and the treatment of those phenolic acids decreased the expression of inflammatory mediators such as IL-6, IL-1-β, tumor necrosis factor (TNF)-α. da Cunha et al. [61] explored the anti-inflammatory capacities of caffeic acid and its derivatives, and their potent inhibition of lipopolysaccharide (LPS)-induced inducible nitric oxide synthase (iNOS) expression in RAW 264.7 macrophage was found. Chtourou et al. [56] evaluated the anti-inflammation capacity of naringin in cisplatin-induced nephrotoxicity in rats and found its effective anti-inflammation capacity. Kamel et al. [57] explored the efficiency of hesperidin and rutin on the attenuation of cisplatin-induced nephrotoxicity in rats; the administration of hesperidin (200 mg/day) or rutin (30 mg/day) for 14 days actively suppressed the inflammation of rats tested. Furthermore, apigenin ameliorated inflammation through the reduction of p53 activation in human renal proximal tubular epithelial cells [55]. In addition, kaempferol modulated pro-inflammatory enzyme activities, gene expression related to inflammation, and inhibition of transcription factor such as NF-κB, showing unusual anti-inflammation activity [52]. Calderon-Montano et al. [54] also reported anti-inflammation potential of kaempferol. Hamalainen et al. [62] investigated anti-inflammation activities of representative flavonoids such as naringenin, quercetin, kaempferol, isorhamnetin, daidzein, and genistein as well as pelargonidin (anthocyanidin). All compounds listed above suppressed iNOS protein by inhibiting nuclear factor-κB (NF-κB), which is a main transcription factor for iNOS. 

### 2.3. Antibacterial and Anti-Viral Activity 

Antibacterial and anti-viral agents kill or slow down the action of bacteria and viruses without inflicting any damage to the surrounding cells and tissues. Up until now, many compounds with the characteristics mentioned above have been found. In this connection, phenolics have also been shown to be potent antibacterial and anti-viral agents. For instance, phenolics constrained the growth and proliferation of hepatitis C virus (HCV); this virus is a primary blood-borne pathogen causing liver cirrhosis and hepatocellular carcinoma (HCC), thus inhibiting infection in primary human hepatocytes [42]. Kang et al. [43] reported that gallic acid and its derivatives attenuate the growth of cariogenic and periodontopathic bacteria. Gallic acid and methyl gallate also exhibited strong antibacterial and antiviral potential against *Salmonella* [44]. Kane et al. [63] stated inhibitory activity of gallic acid and methyl gallate against herpes viruses. They argued that the attachment of these phenolics to the virus proteins may interfere with their invasion of cells. Lin et al. [50] reported the suppression of virus type-1 infection of (+)-epigallocatechin 3-*O*-gallate. Moreover, phenolics such as stilbenes, tannins, and isoflavones inhibited the growth of fungi, yeasts, and viruses as well as bacteria such as *Salmonella*, *Clostridium*, *Bacillus*, and *E. coli* [64,65,66]. 

### 2.4. Other Bioactivities of Phenolics

Aside from anticancer, anti-inflammation, and antibacterial/-virus potential, a variety of clinical trials have established other beneficial bioactivities of phenolics. Ferulic acid alleviated angina pectoris and hypertension and is used as a traditional medicine in China [46]. Additionally, ferulic acid reduces the incidence of type 2-diabetes, cardiovascular disease, and neurodegenerative diseases [34,45,67]. Chlorogenic and caffeic acids inhibit the formation of mutagenic and carcinogenic N-nitroso compounds [47]. Chao et al. [60] tested the effect of caffeic and ellagic acid on the suppression of diabetic kidney diseases of the rat. The intake of those phenolic acids for 12 weeks significantly reduced the levels of urinary glycated albumin, sorbitol, and fructose as well as suppression of sorbitol dehydrogenase activity. Flavonoids have also shown powerful bioactivities. For instance, catechins and their derivatives relieved degenerative diseases and brain aging processes. In addition, they are verified as strong neuroprotective chemicals that are useful for preventing Parkinson’s and Alzheimer’s diseases [48,49,68,69]. Catechin is also an effective agent that retards the development of atheromatous lesion [70]. Kaempferol is a potent neuroprotection agent by attenuating caspases cleavage and apoptotic nuclei, followed by guarding SH-SY5Y cells and important neurons from rotenone toxicity [53]. Trivedi et al. [51] reported the anti-osteoclastogenic effect of kaempferol, as well as its interference in the formation of adipocyte in bone marrow cells (BMCs). Quercetin revealed blood pressure-lowering effect by displaying antioxidant potential, inhibitory activity against angiotensin-converting enzyme (ACE), and enhanced endothelium-dependent and -independent function [71]. Quercetin limits the incidence of coronary heart disease by attenuating the expression of metalloproteinase 1, and they also interfere with the accumulation of plaques in the artery wall [72]. Resveratrol represses cyclooxygenase 1 activity, leading to a reduction in the accumulation of platelets [73], and exhibits anti-diabetes potential by modulating SIRT1, which contributes to the homeostasis of blood sugar content in diabetic rats [74]. Polyphenols such as (+)catechin, (−)epicatechin, isoflavones, tannic acid, and chlorogenic acid have also revealed anti-diabetes potential by reducing the intestinal transport of glucose through S-Glut-1 [75]. The consumption of genistein improved the function of the lung in asthmatic patients [76]. Moreover, isoflavones such as genistein and daidzein enhanced bone mineral density in ovariectomized rats [77]. Theaflavins, which are abundant in black tea, restrained the invasion of HIV-1 cells into the target cells, showing inhibitory activity against HIV-1 infection [78]. Theaflavin derivatives such as theaflavin–digallate and theaflavin–gallate prevented Severe Acute Respiratory Syndrome (SARS) coronavirus by inhibiting the chymotrypsin activity that plays a significant function in viral multiplication [78]. Tsuda et al. [79] stated that anthocyanidins, which are referred to as an efficient modulator of the metabolism of adipose tissue, showed strong potential for reducing obesity, inhibiting dysfunction of fat cells, and attenuating fat accumulation in adipose cells. In addition, anthocyanidins suppressed lipid oxidation and ameliorated the incidence of inflammation by inhibiting cyclooxygenase (COX)-1 and -2 [80]. Tannins exert different health-beneficial effects such as enhancing blood clotting, reducing blood pressure, dropping serum lipid level, producing liver necrosis, and modulating immune responses [81,82].

## 3. Bioactivities of Phenolic Extracts from Natural Origin

### 3.1. Grains/Cereals

Grains are rich in carbohydrates, lipids, and proteins, together with a high level of phytochemicals such as phenolics. Some phytochemicals exhibit direct biological activities, i.e., anticancer, anti-inflammatory, and antibacterial potential, whereas others may function indirectly by acting as the cofactor of antioxidant enzymes. Epidemiological evidence has shown that regular consumption of grains and cereals reduces the incidence of chronic ailments such as vascular disease, type 2 diabetes, and different types of cancer [83,84,85]. In this section, the bioactivities of phenolic extracts of grains and cereals are discussed by focusing on their preventive effects on chronic ailments (Table 2). 

Black rice phenolics which includes a high level of anthocyanidins, namely cyanidin 3-glucoside, malvidin 3-galactoside, peonidin3-glucoside, and pelargonidin 3,5-diglucoside, efficiently alleviated the incidence of chronic diseases such as antiatherosclerosis, antitumor, antiallergic, and, antifatigue activities as well as improving hypoxia tolerance [86,87,88,89]. Chandrasekara and Shahidi [90] found a dose-dependent inhibitory capacity against DNA scission and oxidation of LDL cholesterol for ferulic and *p*-coumaric acids that were isolated from millets. Surendrian et al. [91] reported that wild rice enhanced the activity of antioxidant enzymes such as superoxide dismutase (SOD) and catalase (CAT) due to the presence of phenolics in a mouse model system. Moreover, the ferulic acid in rye resulted in a remarkable decrease of mtDNA 8-OhdG levels in the liver, kidneys, and pancreas in mice [92]. 

### 3.2. Legumes/Seeds

Legumes and seeds are rich in proteins and dietary fiber as well as other nutrients and antioxidant compounds, which leads to their beneficial health effects in preventing aging, strengthening immunity, lowering cholesterol levels, and reducing cardiovascular diseases. Apart from the aforementioned compounds, legumes/seeds contain a high level of phenolics including phenolics acids, flavonoids, and proanthocyanidins, which are responsible for the efficient bioactivities. The hull portion of legumes and seeds possesses a higher content of phenolic compounds than the corresponding dehulled parts due to the presence of phenolic-containing cells such as epidermis, hypodermis, chlorenchyma, palisade, parenchyma, and endothelium cells [21]. The vacuole, which is a key organ of a plant cell, reserves the largest volume of water of the cells along with plant metabolites such as phenolics and organic acids. The high level of phenolics in legumes and seeds contributes remarkable bioactivities to products. For instance, peas contain a variety of phenolics such as gallic acid, epigallocatechin, naringenin, and apigenin, which results in their potential chemopreventive and complementary properties in cancer therapy [94]. Sergent et al. [93] have demonstrated the lipase inhibitory effect of epigallocatechin-3-gallate, kaempferol, and quercetin extracted from grape seeds. Thompson et al. [95] reported that lignans from flaxseed had protective potential against chemically induced carcinogenesis in animal models. Alshikh et al. [15] isolated free, esterified, and insoluble-bound phenolics from selected lentils and these showed inhibitory activity against cupric ion-induced human LDL peroxidation and peroxyl radical-induced DNA strand breakage. In addition, anthocyanins in black soybeans displayed anti-inflammatory activity in a rat model system [96], and the phenolic acids, flavonoids, and anthocyanins of navy and black beans suppressed the mRNA expression of colonic inflammatory cytokines such as IL-6, IL-9, IFN-g, and IL-17A in a mouse model of acute colitis [97]. Ethanol extract of red beans containing catechin-7-β-d-glucopyranoside efficiently inhibited nitric oxide (NO) production in both in vitro and in vivo models [98]. 

### 3.3. Fruits/Vegetables

Fruits contain a high level of phenolics and have shown excellent bioactivities such as anticancer and anti-inflammatory activities. Patterson and Murray [99] have reported that regular consumption of pomegranate juice, which contains punicalagin A, punicalagin B, and ellagic acid, as well as anthocyanins such as cyanidin–diglucoside, pelargonidin, delphinidin–diglucoside, and cyanidin–pentoside, suppresses the growth of cancer cells by reducing total hepatic cytochromes P450 (CYP) content in a mouse model system. In addition, pomegranate juice attenuated inflammatory cytokine expression, followed by an anti-inflammation effect [100]. The chronic consumption of oranges reduced the risk of breast cancer due to the presence of flavonoids [102,103]. Serra et al. [104] found several phenolics such as catechin, procyanidins (B1 and B2), phloridzin, and epicatechin in apples, and these phenolics showed anticarcinogenic potential. Moreover, phenolics in Indian gooseberry such as myricetin, quercetin, gallic acid, ellagic acid, and tannins such as glucogallin (1-*O*-galloyl-β-d-glucose), corilagin, pedunculagin displayed anticancer potential [101]. Phenolics in blackberry such as cyanidin-3-glucoside, cyanidin-3-galactoside, malvidin-3-*O*-arabinoside, and delphinidin derivatives also exhibited anticancer activity [101]. Citrus inhibited the release of pro-inflammatory cytokines such as NO, prostaglandin E2 (PGE2), IL-1b, and TNF-α in RAW 264.7 macrophages model system [105]. Resveratrol, found in grapes and some blueberries, has been proven to enhance cognition in rodents [106]. Moreover, phenolics in strawberries display antioxidant defense by suppressing the NF-κB signalling pathway [107]. Some polyphenols in oranges contribute to preventing cardiovascular disease and inflammation [108]. Thus, fruits have been proven to be excellent foods for preventing and relieving chronic ailments due to their high content of phenolics. 

Vegetables also contain a variety of phenolics; for instance, parsley and celery possess apigenin, chrysin, and luteolin, and broccoli has a considerable amount of quercetin, kaempferol, and myricetin, leading to their potent inhibition of inflammation and different types of cancers such as gastric, breast, and prostate [109].

## 4. Conclusions

In this review, the bioactivities of phenolics as such or present in extracts from natural origin such as grains, cereals, legumes, seeds, and fruits were summarized. A large body of epidemiological evidence exists on their protective effects against a number of chronic ailments such as cancer, inflammatory diseases, and bacterial disorders, as well as reducing diabetes, cardiovascular and neurodegenerative diseases. Moreover, the inhibition of Parkinson’s and Alzheimer’s diseases, as well as anti-analgesic, anti-allergic, cardioprotective, and anti-diabetic activities have also been documented for food phenolics. Thus, it is expected that phenolic compounds serve as useful natural bioactive and nutraceutical agents for preventing/inhibiting adult ailments.

## Figures and Tables

**Table 1:** Bioactivities of phenolic acids and flavonoids.

	Bioactivity	Reference
**Phenolic Acid**		
Gallic acid	Anticancer HCV inhibition Antibacterial	[30,31] [42] [43,44]
Methyl gallate	Anticancer	[32]
*p*-Coumaric acid	Anticancer	[36]
Ferulic acid	Anticancer Alleviates angina pectoris Reducing hypertension Reducing type 2-diabetes	[33,45] [46] [46] [45]
Chlorogenic and Caffeic acids	Anti-mutagenic and-carcinogenic activity	[47]
**Flavonoid**		
Catechin	Preventing Parkinson’s and Alzheimer’s diseases	[48,49]
(+)-Epigallocatechin 3-*O*-gallate	Anti-virus	[50]
Quercetin	Anticancer	[51]
Kaempferol	Anticancer Anti-inflammation Osteoporotic activity	[40,52,53] [54] [53]
Myricetin	Anticancer	[41]
Apigenin	Anticancer Anti-inflammation	[39] [55]
Troxerutin	Anticancer	[38]
Naringin	Anti-inflammation	[56]
Hesperidin	Anti-inflammation	[57]
Rutin	Anti-inflammation	[57]

**Table 2:** Bioactivities of phenolic extract from natural origins. LDL: low density lipoprotein; SOD: superoxide dismutase; CAT: catalase.

	Phenolics	Bioactivities	References
**Grains/Cereals**			
Black rice	Flavones, tannin, and anthocyanidins	Anti-atherosclerosis activity Antitumor activity Anti-allergic activity Anti-fatigue and hypoxia tolerance	[86] [87] [88] [89]
Millet	Phenolic acids, flavonoids, and proanthocyanidins	Inhibition of radical-induced DNA scission and the oxidation of human LDL cholesterol	[90]
Wild rice	Phenolics	Enhancing SOD and CAT activities	[91]
Rye	Ferulic acid	Decrease in mtDNA 8-OhdG levels in liver, kidneys, and pancreas	[92]
**Legumes/Seeds**			
Grape seed	Kaempferol and quercetin	Inhibition of lipase activity	[93]
Pea (seed coats)	Phenolic acids and flavonoids	Anticancer	[94]
Flaxseed	Lignan	Anticancer	[95]
Lentil	Phenolic acids, flavonoids, and proanthocyanidins	Inhibition of radical-induced DNA scission and the oxidation of human LDL cholesterol	[15]
Black soybean	Anthocyanins	Anti-inflammation	[96]
Navy and black bean	Phenolic acids, flavonoids, and anthocyanins	Anti-inflammation	[97]
Red bean	Catechin-7-β-d-glucopyranoside	Anti-inflammation	[98]
**Fruits**			
Pomegranate juice	Phenolic acids, flavonoids, and proanthocyanidins	Anticancer Anti-inflammation	[99] [100]
Blackberry	Phenolic acids, flavonoids, and tannins	Anticancer	[101]
Indian gooseberry	Phenolic acids and flavonoids	Anticancer	[101]
Orange	Flavonoids	Anticancer (breast cancer)	[102] [103]
Apple	Catechin, procyanidins, and phloridzin	Anticancer	[104]
Citrus	Narirutin	Anti-inflammation	[105]
